# Passing Events and Topics

**Published:** 1922-01

**Authors:** 


### PASSING EVENTS AND TOPICS.

ON THE danger of Anthrax in shaving brushes, Dr. E. H. Snell, the Medical Officer of Health of Coventry, writes in his annual report for 1920:—"On May 3 I received a letter from the Medical Superintendent of the Northwood Sanatorium, to the effect that a Coventry patient, named E. C., who was admitted to that Institution on April 12, had developed a "Malignant pustule" on the chin, which, from its situation, seemed to suggest infection with Anthrax from a shaving brush. An operation was immediately performed, and the pustule excised, together with the infiltrated tissues around. At the date of the letter (May 2) no constitutional symptoms had shown themselves; but Anthrax infection is always a very serious condition. It appears that E. C. purchased a new shaving brush prior to leaving for Northwood from Mr. G. Mr. G.'s shop has been visited, and it has been ascertained that during the past three months three different varieties of shaving brushes have been obtained from three different sources, two from London and one from Birmingham. I immediately sent a description of the three varieties to the Medical Superintendent of Northwood, in order to ascertain which particular variety was suspected, and in the meantime Mr. G. has promised to cease the sale of any of these brushes until he hears further. Subsequently ten of these brushes were submitted for bacteriological examination, and two were proved to contain Anthrax spores. The remainder of the brushes were purchased for destruction." The patient made a satisfactory recovery.

---

THE new Home for the domestic staff at the Norfolk and Norwich Hospital is now in occupation. Among other things, it provides a separate bed oom for each maid. Thereby it adds another to the already considerable advantages offered by institutional, over private, domestic service. This Home is one of the first of its kind, and deserves to be widely known. It is an instalment of the fulfilment of our once mocked prophecy, that the time would come when a Home for the domestic staff would be as essential to a modern hospital as a Home for nurses.

---

TO help to raise the £100,000 required upon its 150th anniversary, the Leicester Royal Infirmary has devised an ingenious levy, based upon a "unit system." The unit is 12s. 6d. (150 pennies), and one hundred and fifty thousand units are sought, in proportions of 48 thousand from the county, and 112 thousand from the city, of Leicester, according to the respective proportion of patients treated from either. In the county four residents can club together to provide one unit, and a similar estimate of the city population gives the required share per unit for each resident or family there. At the time of writing we hear that the scheme has received many promises of support.

AT a meeting of the Society for the Prevention of Cruelty to Children which took place recently in Dublin, Sir Andrew Horne made the statement that baby farming, accompanied by heavy infant mortality was being carried on in Ireland. He instanced the case of one woman who took babies for money, and during ten years had buried 24 out of 25 of the infants that she received. Public health conditions have never been very flourishing in that most distressful country, but we hope that under the new regime much more effort and money will be spent in ameliorating the condition of the Irish children. If the new Free State is to be happy and successful it must first improve the general condition under which the people exist, and safeguard also the health of the future generation. Unhealthy people are not happy; and a miserable and dissatisfied Ireland will never be able fully to take her right place among the nations.

---

"NO financial worries" is the present boast of North Ormesby Hospital, Middlesbrough, which has lately opened a new X-ray department, costing £1,200, under Dr. Howell. Mr. J. H. Amos, a member of the Hospital Council, who is also manager of the Tees Conservancy Board, collected almost the whole sum.

---

IT is well that logic was weak in the testator who transferred a substantial legacy from hospitals, on the ground that "it would benefit the majority of the loafers, formerly called workers," to the Royal National Pension Fund for Nurses, "as nurses, unlike three-fifths of the loafing wastrels generally, have never wantonly and even maliciously gone on strike without the slightest provocation, sense, or justification." No one will quarrel with this tribute, and an admirable institution benefits; but a person who objected to providing medical aid for even the innocent dependents of "wastrels" might have been expected to remember that nurses are occasionally guilty of selecting their fathers from that class. And in despair of finding a worthy object he might have left the money to the Bolshevik Bedsocks Fund.

---

A correspondent in a Scotch paper discusses the suitability of the London National Provident Scheme for Hospital and Additional Medical services to Scotland, and particularly to Edinburgh. He states that the Committee of the Royal Infirmary, Edinburgh, might not approve the contractual element in the London Scheme, because, he suggests, the immense waiting lists plus the "legal right" to a bed conferred by the scheme (with safeguards, however) might create difficulty in the northern capital. He urges none the less careful consideration of the plan, and suggests a conference of delegates from the Royal Infirmary, the Eye, Ear and Throat Infirmary, the Hospital for Diseases of Women, and the Royal Edinburgh Hospital for Sick Children, to meet men with actuarial and insurance experience. We hope that his suggestion will be adopted.

**PASSING EVENTS AND TOPICS—(*cont.*).**

WRITING of rat and mice destruction in his annual report for 1920 on the sanitary condition of the Borough of Poplar, Dr. F. W. Alexander, the Medical Officer of Health, says: "During the organised 'Rat Weeks' a large quantity of barium carbonate paste was distributed to applicants, the results being highly satisfactory. Lithographic varnish traps were also used with success. The number of complaints of the presence of rats and mice has greatly diminished. It must be added that *every week is a 'rat week'* in Poplar, and the work of destruction proceeds, principally by the use of barium carbonate paste which is still being distributed to tenants of houses, where necessary, and is recommended for use in factories, etc. Where rats are still persistent after the use of poison the ground is opened and the drain examined as a matter of routine. Accumulations of refuse are also directed to be removed."

---

DANCING maintains its popularity. Like roller-skating, it has a vogue about every five years. Hospitals are trying to make the most of it this winter, and "balls right through the winter" are being discussed. The Great Northern Central Hospital held what may be the first of a series on December 15 at the Hyde Park Hotel, under the guidance of Lady Newnes and Lady Sargant, R.R.C., the latter of whom has had some training in nursing. Among the poorer classes, especially in the provinces, dancing, and particularly whist drives, are often weekly events. The organisers have only to be approached by secretaries of the local hospital to find, we believe, a new and constant source of interest and revenue in these affairs.

---

THE "Pall Mall and Globe" recently published the following:—

> We are getting tired of the wail of the London hospitals, with their frantic appeals to a public which is waiting for the accommodation the hospitals refuse to supply.
>
> Will these institutions understand once and for all that they must put themselves upon a paying basis and cater for a public willing and anxious to pay reasonable rates for nursing and medical attention as well as for the public that cannot pay?
>
> Until every hospital establishes paying wards for paying patients the middle-class public may be advised to keep their money in their pockets; they will need it for their own treatment in extortionate nursing homes.
>
> If the hospitals will not do this, the obviously right thing, then they will pass into the hands of the State. There is no alternative.

Our contemporary, it would appear, would wish our hospitals to reorganise themselves on American lines. Hospitals in America are business propositions and profitable ones at that. American hospitals are very popular, and, of course, are admirably managed; and, as with hotels in this country, their charges are graduated to suit the means of all classes of patients except the very poor. To convert our present hospitals in a twinkling into institutions similar to those in America is neither immediately possible nor desirable; but we should watch with great interest the adaptation of one of our smaller hospitals.

THIS year has been an exceptional one in the history of the Gloucestershire Royal Infirmary and Eye Institution. The centre block and north wing have been connected on the first floor by a fire-proof bridge, and an electric lift has been installed—so that patients from the wards are now taken to the operating theatre without the discomfort of being carried up and down stairs, and patients coming in to the hospital are sent straight to any ward with far less disturbance than formerly. The hospital is indebted to the Mayoress, Mrs. Roberts, for these alterations. Her efforts to raise funds for the purpose have been untiring and her personal interest in the institution is both cheering to the staff, and stimulating to the public. At the formal opening in the presence of a large band of her helpers from the city and county, the Mayoress formally opened the bridgeway with a presentation silver gilt key, suitably inscribed, and there emerged from the lift, on its first official journey, a little patient who presented Mrs. Roberts with a beautiful bouquet. Tea was served in one of the wards, and the visitors afterwards inspected the new kitchens—another recent addition, for which it is indebted to Red Cross funds.

---

WE much regret to hear of the death, at the age of 80, of Sir Perceval Alleyn Nairne, of 176, The Grove, Camberwell, senior partner in the firm of Baker & Nairne, solicitors, of Crosby Square. He had served on the committee of management of the Seamen's Hospital for fifty-two years, and was the oldest hospital chairman in London. Sir Perceval was for twenty-five years hon. secretary to the Rochester Diocesan Conference, and he was a keen yachtsman and freemason.

---

SIR ARTHUR PEARSON'S death is a loss, not only to the blind and to those who work for them, but to the whole community, which can never be too rich in examples of courage. Regret for the cutting short of a valuable life is deepened by the tragic nature of its ending. Many of the blinded men who passed through St. Dunstan's owe him, not only their admirable training, but the restoration of hope and interest in life by the mere fact of his active and cheerful presence in the darkened world he shared with them.

